# TLR-3 receptor activation protects the very immature brain from ischemic injury

**DOI:** 10.1186/1742-2094-10-104

**Published:** 2013-08-21

**Authors:** Hui Shi, Nadia Gabarin, Edward Hickey, Rand Askalan

**Affiliations:** 1 Program of Neuroscience and Mental Health, Research Institute, Hospital for Sick Children, Toronto, Ontario M5G 1X8, Canada; 2 Department of Surgery, Hospital for Sick Children, Toronto, Ontario M5G 1X8, Canada; 3 Department of Pediatrics, University of Toronto, Toronto, Ontario Canada; 4 Division of Neurology, Hospital for Sick Children, Toronto, Ontario M5G 1X8, Canada

**Keywords:** Preconditioning, Neonatal stroke, Toll-like receptors, Neuroinflammation, Neuroprotection, Premature brain, Hypoxic-ischemic injury

## Abstract

**Background:**

We have shown that preconditioning by lipopolysaccharide (LPS) will result in 90% reduction in ischemic brain damage in P7 rats. This robust LPS neuroprotection was not observed in P3 or P5 pups (corresponding to human premature infant). LPS is a known Toll-like receptor 4 (TLR-4) ligand. We hypothesized that TLRs other than TLR-4 may mediate preconditioning against cerebral ischemic injury in the developing brain.

**Methods:**

TLR-2, TLR-3, TLR-4, and TLR-9 expression was detected in brain sections from P3, P5, and P7 rats by immuno-staining. In subsequent experiments, P5 rats were randomly assigned to TLR-3 specific agonist, poly I:C, or saline treated group. At 48 h after the injections, hypoxic-ischemic (HI) injury was induced by unilateral carotid artery ligation followed by hypoxia for 65 min. Brains were removed 1 week after HI injury and infarct volumes were compared in H&E stained sections between the two groups.

**Results:**

TLR-2 and TLR-3 were highly expressed in brains of P3 and P5 but not in P7 rats. The number of TLR-4 positive cells was lower in P3 and P5 compared to P7 brains (*P* <0.05). TLR-3 was predominately expressed in P5 pups (*P* <0.05). There was no significant difference in TLR-9 expression in the three age groups. There was a significant reduction in infarct volume (*P* = 0.01) in poly I:C compared to saline pre-treated P5 pups. Pre-treatment with poly I:C downregulated NF-κB and upregulated IRF3 expression in P5 rat ischemic brains. Pre-treatment with poly I:C did not offer neuroprotection in P7 rat brains.

**Conclusion:**

TLRs expression and function is developmentally determined. Poly I:C-induced preconditioning against ischemic injury may be mediated by modulation of TLR-3 signaling pathways. This is the first study to show that TLR-3 is expressed in the immature brain and mediates preconditioning against ischemic injury.

## Background

Toll-like receptors (TLRs) are a family of transmembrane pattern recognition receptors that play a key role in host defense against pathogen infection. These receptors recognize a variety of pathogen-associated molecular patterns (PAMPs), such as lipopolysaccharide, peptidoglycan, bacterial DNA, and double-stranded RNA [1]. There are 13 mammalian TLRs with TLRs 1 to 9 being conserved between humans and mice. The expression of TLRs and their role in inflammation and ischemic injury in the adult brain is well documented. TLR-4 expression has been observed in the meninges, choroid plexus, and circumventricular organs of the adult rat brain [2]. In the human CNS, microglia express TLRs 1 to 9, astrocytes express robust TLR-3 and low-level TLRs 1,4,5,9 and oligodendrocytes express TLR-3 and TLR-2 [3,4]. Cerebral ischemia results in increased TLR-4 and TLR-2 expression in the brains of adult mice [5]. Furthermore, mice deficient in TLR-4 and TLR-2 display reduced infarct size after ischemic injury compared to wild-type mice [5]. Taken together, these results indicate the TLRs play an important role in ischemia-induced injury in the adult brain.

While there is accumulating knowledge on the expression and function of TLRs in the adult CNS, little is known about TLRs in the developing brain. TLR-8 and TLR-3 are expressed in neurons of embryonic and neonatal mouse brains where they regulate neuronal growth [6,7]. We have shown that TLR-4 is expressed in postnatal day 7, 9, and 14 rat brains [8]. More recent studies have shown that TLRs 1 to 9 are expressed in the P9 mouse brain [9]. Cerebral ischemia has been shown to increase the expression of a number of TLRs in neonatal mice [9]. However, the role of TLRs in ischemic injury of the developing brain is yet to be determined.

Ischemic tolerance or preconditioning is a phenomenon by which a sub-injurious stimulus is applied to a tissue such as the brain. After a certain delay, the brain develops tolerance to ischemic injury caused by the injurious stimulus. Ischemic preconditioning, therefore, protects against subsequent ischemic injury. The delay to protection may be minutes to few hours (rapid or early preconditioning) or days (delayed preconditioning) requiring protein synthesis [10-12]. Since Kitagawa and colleagues first reported on delayed preconditioning in 1991 [13], this phenomenon has been well documented in the brain. Although brief cerebral ischemia or hypoxia is the typical ischemic preconditioning stimulus [14], ischemic preconditioning may also be induced by exposing the brain to a variety of stimuli such as inflammation, oxidative stress, hyperthermia, and spreading depression [11,12]. Activation of TLR2 and TLR9 by their highly specific ligands (Pam3CSK4 and unmethylated CpG ODN, respectively) has been shown to induce ischemic preconditioning in adult stroke models [15,16]. We have recently shown a robust delayed preconditioning against ischemic injury in the neonatal rat [8] and piglet [17] brains induced by lipopolysaccharide (LPS), a TLR-4 specific agonist.

We reported that LPS-induced neuroprotection against cerebral ischemic injury was offered to P7, P9, and P14 rat pups. LPS neuroprotection was ineffective in P3 and P5 rat pups, and the brains of these pups expressed significantly less TLR-4 compared to P7, P9, and P14 rats [8]. In light of these findings, we sought in this study to investigate the effect of brain maturity on TLRs expression and to examine whether TLRs other than TLR-4 offer neuroprotection to the developing brain against cerebral ischemic injury. We chose TLR-2 and TLR-9 because of their potential capability of mediating preconditioning in the rat immature brain given their neuroprotective effect in adult brain and heart [15,16,18]. We also examined the expression of TLR3 because it is the only receptor that share MyD88-indpendent signaling pathway with TLR4. It is plausible that TLR3 has a neuroprotective function specific to the developing brain independent of TLR4.

## Methods

### Immunohistochemistry

#### TLRs expression

Brains from rat pups aged P3, P5, and P7 (*n* = 6 rats for each age group) were removed and immediately fixed in 10% formalin. Paraffin-embedded coronal sections were cut (8 μm) at the level of the dorsal hippocampus, de-waxed with xylene, hydrated, and pre-treated with heat-induced antigen retrieval technique. Sections were then stained with rabbit anti-TLR-4 (1:50; Santa Cruz), rabbit anti-TLR-3 (1:50; Santa Cruz), rabbit anti-TLR-2 (1:50; Santa Cruz), rabbit anti-TLR-9 (1:50; Santa Cruz) at 4°C overnight. TLRs expression was then detected by goat anti-rabbit horseradish peroxidase (HRP; 1:100; Chemicon) for 1 h at room temperature and DAB substrate kit for Peroxidase/Vector/SK-4800. The number of TLRs positive cells were counted in four to five high power fields (40×) using Image J computer software (National Institutes of Health, Bethesda, MD, USA) and compared among the different age groups.

#### Cellular localization of TLR-3 expression

Antibody for CD68, glial fibrillary acidic protein (GFAP), neuronal nuclei (NeuN), and O4 are well-established markers for detecting microglia, astrocytes, neurons, and oligodendrocytes, respectively. To determine whether microglia, astrocytes, neurons, and/or oligodendrocytes in P5 developing brain express TLR-3, frozen brain sections were incubated simultaneously with TLR-3 antibody (1:50, Santa Cruz) and mouse anti-CD68 (1:50, Santa Cruz), mouse anti-GFAP (1:500, Sigma Chemicals Co), mouse anti-NeuN, (1:100, MAB377, Chemicon), or mouse anti-O4 (1:200, Sigma Chemicals Co). Immuno-reactivity was visualized using appropriate combinations of goat anti-rabbit Fitc (1:200, Jackson Lab) and goat anti-mouse Cy3 (1:200, Santa Cruz) secondary antibodies and nuclei were counterstained with DAPI (Sigma). Multichannel images were captured and analyzed with Nikon NIS-Element Basic Research Image system.

### Treatment protocol for TLR-3 agonist

Pairs of pregnant Wistar rats (Charles River Laboratory) underwent natural delivery of their litters within our animal research laboratory. Pups from each litter (3 litters/age) were randomized to intra-peritoneal injection of Polyriboinosinic:polyribocytidylic acid (poly(I:C)), a stable synthetic dsRNA analogue that has been extensively used as a TLR-3 specific agonist [19-21] or normal saline injection (*n* = 9-11 pups/group/age) and nursed together with their dam. At postnatal day 5 (P5) or 7 (P7) and 48 h before the HI insult, each litter was blindly injected with either 0.3 mg/kg of poly I:C (InvivoGen, San Diego, CA, USA) or an equivalent volume of saline placebo. After injection, animals were returned to a warmed incubator and housed with their dam. The study was undertaken with full approval of the Research Ethics Board and Animal Research Department at The Hospital for Sick Children.

### Hypoxic-ischemic (HI) insult

We used the Rice-Vannucci model, the most commonly used model to study HI brain injury in the developing brain [22]. Rats (Wistar) aged P5 or P7 correspond to pre-term or term human newborn, respectively [23]. Unilateral internal carotid artery ligation in rat pups followed by exposure to 8% hypoxia for approximately 65 min causes a reproducible unilateral infarct ipsilateral to the ligated artery involving caudate, putamen, hippocampus, and cortex [24]. Body temperature was maintained at 37-37.5°C during hypoxia using an incubator. Animals were then killed at 1 week after the HI insult using pentobarbital. Brains were removed and processed to be used in hematoxylin and eosin (H&E), immunohistochemistry, and western blot studies.

### Measurement of infarct volume

Brains were immediately removed 1 week after the HI insult, fixed, embedded in paraffin, and cut into 5 μm coronal sections. The total area of brain tissue loss was measured on H&E stained sections using the Nikon NIS-Element Basic Research Image analysis software system, version 3.0. Total infarct volume in the whole affected hemisphere was calculated according to the Cavalieri principle as we described previously [8].

### Western blots

Rat brains were rapidly removed on a bed of ice, the two hemispheres were separated and homogenized in ice cold homogenizing buffer and then solubilized to be separated by gel electrophoresis. Proteins were then transferred to nitrocellulose membrane to be incubated with anti-NF-κB (1:3,000, cell signaling) or anti-IRF3 (1:1,000, Cell Signaling) at 4° overnight. The membranes were then washed and incubated with goat anti-rabbit horseradish peroxidase antibody (1:5,000; Santa Cruz) at room temperature for 1 h. Immunoreactive bands were visualized using FluorChem FC2 (Alpha Innotech).

### Statistical analysis

All statistical analysis was performed with GraphPad Prism 4 software (Version 4.03; GraphPad software, Inc., San Diego, CA, USA). Comparison of continuous data between experimental groups was made using unpaired *t* test. Normality was evaluated by the Kolmogorov-Smirnov test. All values are presented as mean ± standard error of the mean (SEM). Results were considered to be statistically significant if two-tailed *P* value was ≤0.05.

## Results

### TLRs expression is modulated by brain maturity

**Figure 1:**
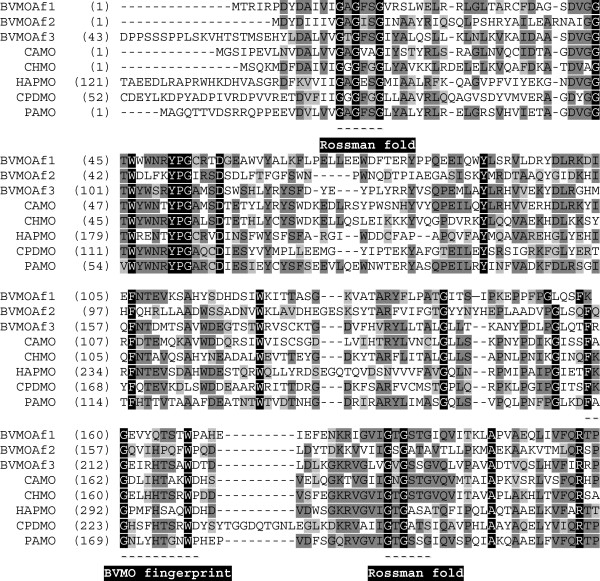
**TLRs expression is determined by brain maturity. (a)** Brain sections from P3 (first column), P5 (second column), and P7 (third column) old rat pups were processed for TLR-2 (top panel), -3 (middle panel), and -4 (bottom panel) immuno-staining as described in Methods. There was a higher cortical expression of TLR-3 in P5 rat brain compared to P3 and P7 whereas TLR-4 was highly expressed in P7 rat brain. Brown color (arrow) denotes positive cell. **(b)** Number of TLRs positive cortical cells was counted by blinded investigator in at least four high power microscopic fields (40×) in each specimen and compared between age groups. ^*^P <0.05 when compared to P7. ^ξ^*P* <0.05 when compared to the other two age groups. There was no significant difference in TLR-9 expression among the three age groups (*n* = 15-30 pups/age group).

In this study, we examined the expression of TLR-2, TLR-3, TLR-4, and TLR-9 in the brains of P3, P5, and P7 rat pups. Our results show a differential expression of TLRs that is determined by brain maturity. As shown in Figure 1a, TLR-2 and TLR-3 were minimally expressed in the brains of P7 rats compared to P3 and P5 rat pups. In fact, TLR-2 and TLR-3 cortical expression were significantly higher in P3 and P5 compared to P7 rats, with the highest expression of TLR-3 in P5 rats (*P* <0.05; Figure 1b). TLR-4 was predominately expressed in P7 rats with minimal expression in the other two age groups. There was no significant difference in TLR-9 expression among the three age groups.

### TLR-3 is expressed in neurons of a normal P5 rat brain

**Figure 2:**
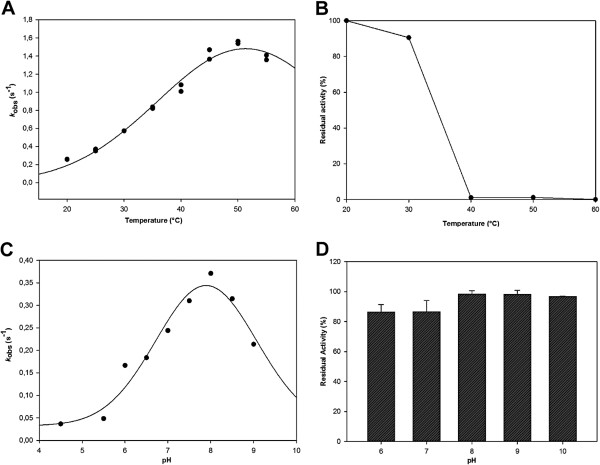
**TLR-3 is expressed in the very immature rat brain.** Double staining with anti-TLR-3/anti-NeuN, a neuronal marker **(a, d)**, anti-TLR-3/anti-GFAP, a marker for astrocytes **(b, e)** and anti-TLR-3/anti-CD68, a marker for microglia **(c, f)** was performed in normal **(a-c)** and ischemic **(d-f)** P5 rat brain as described in Methods. Immuno-detection was performed using immuno-fluorescence staining method (*n* = 6-10 pups/marker); TLR-3 in yellow, NeuN in red **(a)** and green **(d)**, GFAP in brown and CD68 in red. Arrows indicate the co-localization of TLR-3 and NeuN, GFAP, or CD68 in the cortex at the dorsal hippocampal level.

Double staining for TLR-3/NeuN, TLR-3/GFAP, TLR-3/CD68, and TLR-3/O4 revealed that TLR-3 is expressed in neurons of a normal P5 rat brain with minimal expression in astrocytes and absence of expression in microglia (Figure 2a-c) and oligodendrocytes (data not shown). Once we challenged the brain with hypoxic-ischemic (HI) injury, TLR-3 expression increased in astrocytes, increased minimally in microglia (Figure 2d-f) and remained absent in oligodendrocytes.

### TLR-3 activation reduces ischemic damage in P5 rat brain

**Figure 3:**
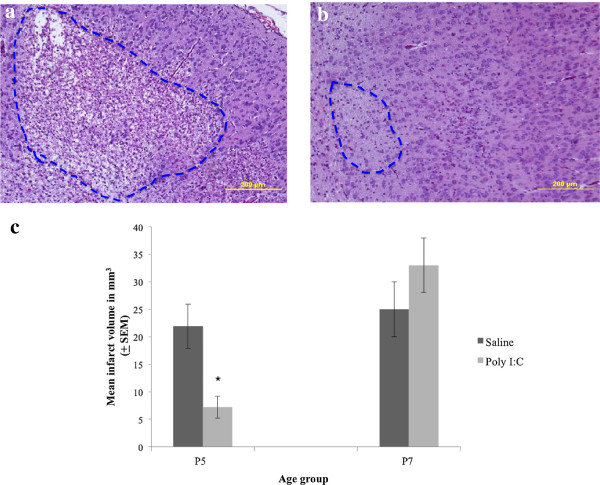
**Preconditioning with TLR-3 agonists reduced infarct volume in P5 ischemic rat brain.** P5 or P7 rats randomly received either a normal saline or poly I:C injection (*n* = 9-11 pups/group/age) as described in Methods. 48 h after poly I:C or normal saline injection, HI injury was induced and animals were then killed 1 week later and brains were immediately removed to assess tissue damage using H&E staining as described in Methods. The area of tissue damage (surrounded by dotted line) in the affected hemisphere is significantly greater in the untreated **(a)** compared to the poly I:C treated **(b)** P5 rat. Infarct volumes were compared between poly I:C treated and untreated P5 and P7 rat pups. The total area of brain tissue loss was measured on H&E stained sections using the Nikon NIS-Element Basic Research Image analysis software system, version 3.0. Total infarct volume was calculated using the formula V = ∑APt, where V is the total volume expressed as mm^3^, A is the sum of the areas measured, P is the inverse of the section sampling fraction (1/200), and T is the section thickness (5 μm) [25]. Poly I:C ischemic preconditioning reduced infarct volume from 21.9 ± 4.0 (mean ± SEM) mm^3^ to 7.1 ± 2.0 (mean ± SEM) mm^3^ in P5 rats but had no protective effect in P7 rats **(c)**. * *P* ≤0.002 when infarct volume compared to saline treated rats of the same age group.

Our results so far have shown that TLR-3 is highly expressed in P5 rat developing brain and it is up regulated as a result of ischemic injury. It is conceivable that TLR-3 activation may induce preconditioning against ischemic injury in the P5 rat brain. To test this hypothesis, we treated P5 rats with TLR-3 specific agonist poly I:C 48 h prior to inducing HI injury and sacrificed the animals 1 week post injury as described in Methods. Pre-treatment with poly I:C reduced ischemic brain injury (Figure 3a,b) and resulted in a 70% reduction in infarct volume compared to saline treated P5 rats (*P* <0.002; Figure 3c). Pre-treatment of P7 rats with poly I:C did not offer protection against ischemic injury (Figure 3c) when compared to untreated P7 rats (*P* = 0.31).

### Preconditioning may be mediated by modulation of TLR-3 signaling pathways

**Figure 4:**
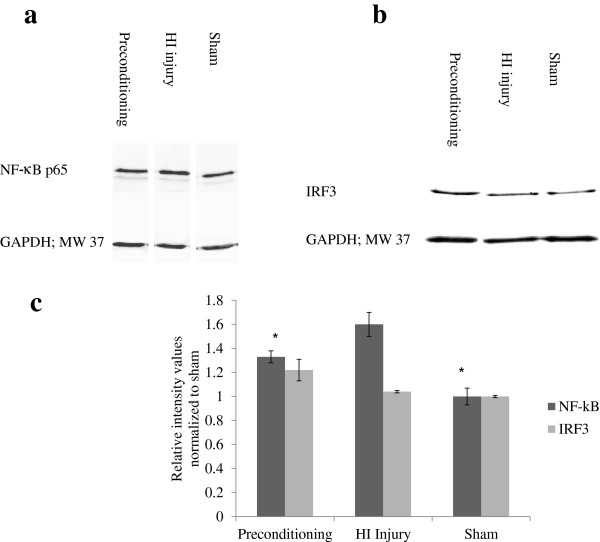
**Preconditioning against ischemic injury may be mediated by modulation of TLR-3 signaling pathways in P5 rat brain.** Western blot analysis performed on brain homogenates from P5 rats pre-treated with 0.3 mg/kg poly I:C 48 h prior to HI injury (Preconditioning), P5 rats with HI injury, and P5 rats with normal brain (Sham) as described in Methods. Representative western blots are shown for NF-κB (**a**; *n* =3) and IRF3 (**b**; *n* =3) expression. Sham relative intensity values were normalized to 1. There was an increase in relative intensity values of NF-κB immune-reactive bands in ischemic rat pups compared to preconditioned (*P* = 0.03) and sham (*P* = 0.008) animals **(c)** whereas there was a trend of increased relative intensity of IRF3 immuno-reactive band in the preconditioned rat pups compared to ischemic and sham rats (*P* = 0.07; **(c)**. GAPDH = Glyceraldehyde-3-phosphate dehydrogenase. *P <0.05 when compared to animals with HI injury.

Once stimulated, TLR-3 is known to activate TIR domain-containing adaptor inducing IFN-β (TRIF) adaptor protein that in turn recruits several transcription factors including NF-κB and IRF3 leading to the production of IL-12 and IFN-β, respectively. Western blot analysis revealed that there was an increase in NF-κB expression in the ischemic P5 rat brain compared to sham animals (*P* = 0.008; Figure 4a,c). This increase was partially reversed in pups pretreated with poly I:C prior to the HI injury (Figure 4a). IRF3 expression, on the other hand, was not affected by HI injury alone but there was a trend of upregulation in the poly I:C pretreated rats (Figure 4b) compared to untreated rats with HI injury (*P* = 0.07; Figure 4c). These results suggest that poly I:C-induced preconditioning may be mediated by differential regulation of TLR-3 signaling pathways.

## Discussion

Many studies showed that low doses of LPS provided neuroprotection when administered 24 h prior to ischemic injury in adult stroke models [26-29]. Fewer studies demonstrated the same LPS-induced neuroprotection in neonatal stroke models [17,30]. Recently, we found that LPS administered 48 h prior to HI injury reduced brain damage and infarct volume by 90% at 1 week post injury [8]. This robust LPS-induced neuroprotection was observed in P7, P9, and P14 rat pups. However, LPS was ineffective in protecting P3 and P5 rats from HI injury [8].

The mechanism of how an inflammatory stimulus, such as LPS, induces preconditioning against ischemic injury is still being investigated. The requirement of *de novo* protein synthesis and the induction of pro-inflammatory cytokines such as TNFα, IL-1β, and IL-6 are believed to be essential for achieving preconditioning [31-33]. The dependency of this neuroprotective phenomenon on pro-inflammatory cytokines raises the possibility that a preconditioning dose of LPS may activate TLR-4 causing a mild inflammatory response that will trigger the expression of negative feedback inflammatory inhibitors including the TLR-4 signaling inhibitors (for example, phosphatidylinositol 3-kinase). These endogenous inhibitors are only induced in response to TLRs or cytokine receptors activation (*de novo* protein synthesis) and will remain upregulated until the subsequent ischemic insult occurs. At this state of suppressed innate immunity, the ischemic injury will be unable to elicit an inflammatory response, resulting in reduced brain damage [34-36]. Recent studies have shown that LPS-TLR4 are not the only mediators of preconditioning against ischemic injury. Neuroprotection can also be achieved by activating TLR-2 and TLR-9 in the adult ischemic mouse brain [16,37].

Studies on TLRs expression in the developing brain are scarce. Protein expression of TLR-8 and TLR-3 has been shown during embryonic development [6,7]. More recently mRNA expression has been detected for all TLR1-9 and regulated by HI injury in neonatal (P9) mouse brain [9]. We have shown high expression of TLR-4 in P7, P9, and P14 but low expression levels in P3 and P5 rat pups [8]. To identify TLRs that may play a role in preconditioning the very immature brain (rat pups aged < P7), we investigated the effect of brain maturity on TLR-2, TLR-3, and TLR-9 expression because of their potential role in neuroprotection in the adult brain. TLR-2 and TLR-3 were highly expressed in P3 and P5 compared to P7 rat pups. These results, taken together, indicate that TLRs expression is developmentally determined.

TLRs are expressed in a variety of cell types including brain cells. Using *in vitro* studies, several laboratories have shown that human microglia and astrocytes express TLR mRNAs [3,4,38]. Microglia of corpus callosum and cerebellum in neonatal rats express TLR-4 and this expression has been shown to be upregulated after hypoxia [39]. This is similar to what we reported here on the increase of TLR-3 expression in microglia after HI injury. Recent studies have also shown that cultured rodent [5] and human [40] neurons express TLR-2, TLR-3, and TLR-4. TLR-2 is also expressed in neurons of neonatal mice and its activation seems to contribute to the HI injury [9]. We have shown here *in vivo* that TLR-3 is expressed in neurons of P5 rat brain. These results indicate that neurons have the capacity to contribute to the ischemia-induced inflammatory response in the developing brain.

The highest expression of TLR-3 is in the P5 neonatal rat brain making it the most likely candidate to induce preconditioning against ischemic injury in this age group. Indeed, pre-treating P5 pups with poly I:C, TLR-3 specific agonist, resulted in a significant reduction in infarct volume. This reduction in brain damage was not observed in P7 pre-treated pups indicating that the neuroprotective effect of TLR-3 receptor activation is age specific. TLR-3 activation has been shown to reduce proliferation of adult human cultured astrocytes and to promote neuronal survival in cultured human brain slices by inducing the expression of neuroprotective mediators and modulating the inflammatory response [41]. There is emerging evidence that TLR-3 is expressed in embryonic brain cells where it plays a role in regulating neurogenesis in the developing mouse brain [7]. To our knowledge, this is the first evidence of a neuroprotective role of TLR-3 against ischemic brain injury.

Stimulation of TLR-3 by poly I:C recruits TRIF, the key adaptor protein in TLR-3 signaling pathways. Recruitment of TRIF leads to the activation of several transcription factors including IRF3 and NF-κB (for review 42). Our data showed that exposing P5 pups to HI injury increased NF-κB expression compared to normal rats. This increase was reversed in P5 rats pre-treated with poly I:C. HI injury alone, on the other hand, did not modulate IRF3 expression. An increase in IRF3 expression was only seen when P5 pups were pre-treated with poly I:C prior to HI injury. Activation of NF-κB and IRF3 results in subsequent production of IL-12 and IFN-β, respectively [42]. IL-12 is a known pro-inflammatory cytokine whereas IFN-β is shown to have anti-inflammatory and neuroprotective effects in adult stroke model [43,44]. We hypothesize, therefore, that TLR-3-induced preconditioning is mediated by upregulation of IRF3 anti-inflammatory pathway and concurrent downregulation of pro-inflammatory NF-κB pathway. We are currently investigating this potential mechanism of TLR-3-induced preconditioning using NF-κB and IRF3 knockout mice.

Preconditioning is not only observed in animals and *in vitro* studies; this phenomenon may occur in the human brain. Several studies have reported that stroke patients with previous transient ischemic attacks had milder neurological deficit at presentation and better outcome [45,46]. The challenge remains in determining how to utilize this phenomenon in a new paradigm that will provide prophylactic therapy for patient populations at high risk of brain ischemic injury, such as children with congenital heart disease. From these children, 1:185 will have a stroke within 72 h of their cardiac procedure that will leave 72% of them with neurological deficit [47]. Preconditioning has the potential to protect patients at high risk of brain ischemic injury from devastating neurological outcome and improve their quality of life. However, we still need to understand the pathways leading to preconditioning to achieve this goal.

## Conclusions

TLRs expression and function are developmentally determined. TLR-3 activation induces preconditioning against ischemic injury in the very premature brain. This neuroprotection is mediated by modulation of TLR-3 signaling pathways. The results of this study will be utilized to design neuroprotective therapies for premature infants who are at known high risk of hypoxic-ischemic brain injury.

## Abbreviations

GFAP: Glial fibrillary acidic protein; HI: Hypoxic-ischemic; P: Postnatal; LPS: Lipopolysaccharide; Poly I:C: Polyriboinosinic:polyribocytidylic acid; TRIF: TIR domain-containing adaptor inducing IFN-β; TLRs: Toll-like receptors.

## Competing interests

The authors declare that they have no competing interests.

## Authors’ contributions

RA designed the study, analyzed the data, interpreted the results, and prepared the manuscript. HS performed experiments and acquired the data. NG performed experiments and prepared the manuscript. EH reviewed and discussed the manuscript. All authors have read and approved the final version of the manuscript.
